# Public health round-up

**DOI:** 10.2471/BLT.26.010326

**Published:** 2026-03-01

**Health emergencies and crisis appeal**

The World Health Organization (WHO) has launched its 2026 global appeal, seeking nearly 1 billion United States dollars to deliver health services to people affected by humanitarian emergencies worldwide. In 2025, WHO-supported operations reached 30 million people, including vaccinations for 5.3 million children, 53 million health consultations, support for over 8000 health facilities and 1370 mobile clinics. The new appeal comes amid rising needs driven by protracted conflicts, climate impacts and recurring disease outbreaks, even as global humanitarian funding continues to shrink.

**Figure:**
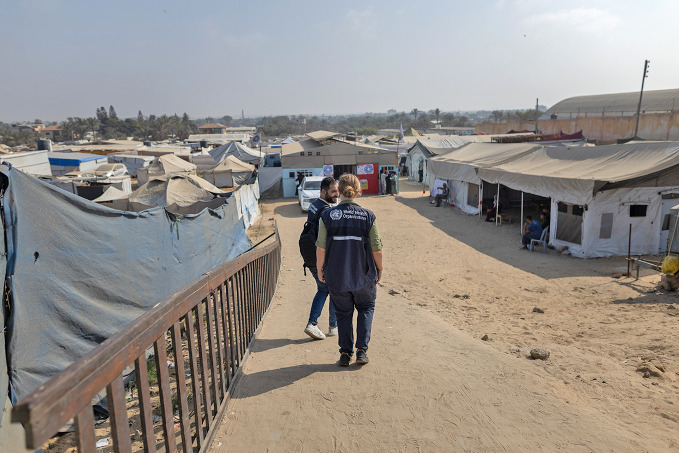
WHO

## Novel oral polio vaccine

The World Health Organization (WHO) has prequalified a new formulation of the novel oral polio vaccine type 2 (nOPV2), expanding global supply of a vaccine essential to stopping type 2 poliovirus outbreaks and advancing eradication efforts. Prequalification confirms the vaccine meets international standards for quality, safety and efficacy, enabling procurement through United Nations (UN) agencies for use across diverse country settings. The newly listed product is manufactured by Biological E. Limited (BioE), India, following a technology transfer from PT Bio Farma (Persero), Indonesia and adds to earlier WHO listings of nOPV2 produced by both companies.

By broadening the manufacturing base, WHO aims to ensure a more resilient and sustainable supply of nOPV2, which was designed to be more genetically stable than the traditional monovalent type 2 vaccine, reducing the risk of seeding new outbreaks while maintaining strong effectiveness. 

Highlighting progress, WHO Director-General Tedros Adhanom Ghebreyesus noted, “Vaccines are also bringing us closer to the eradication of polio, with 41 cases of wild polio reported last year from just 24 districts in Pakistan and Afghanistan, down from 99 cases in 49 districts in 2024.”

The expanded supply supports rapid outbreak response and strengthens global efforts to eliminate polio.

https://bit.ly/4rqcX6R

## Pandemic agreement negotiations continue

The fifth meeting of the Intergovernmental Working Group (IGWG) brought Member States together from 9–14 February to negotiate draft text aimed at ensuring rapid global sharing of pathogen samples and genetic sequence data; critical for accelerating diagnostics, treatments and vaccines during future pandemics. 

The *Pathogen access and benefit sharing* annex, a central element of the WHO *Pandemic agreement* adopted last year, is designed to guarantee that benefits arising from shared materials are distributed rapidly, fairly and equitably.

Delegates reported steady progress, with IGWG Bureau co‑chair Ambassador Tovar da Silva Nunes of Brazil, noting, “Countries this week have again shown their steadfast commitment to getting the *Pathogen access and benefit sharing* annex done.” Co‑chair Matthew Harpur added that while differences remain, “the coming weeks will be critical in bridging the remaining gaps and delivering a *Pathogen access and benefit sharing* annex that is fair, effective and fit for purpose.”

WHO Director‑General Tedros Adhanom Ghebreyesus expressed confidence that Member States will finalize the annex ahead of the World Health Assembly in May this year.

https://bit.ly/4rVPKJq

## UN joint statement on female genital mutilation 

UN leaders marked the international day of zero tolerance for female genital mutilation on 5 February with a joint call to accelerate global efforts to end the practice, warning that 4.5 million girls are at risk in 2026 alone and more than 230 million women and girls already live with its lifelong consequences. 

The heads of the United Nations Population Fund (UNFPA), the United Nations Children's Fund (UNICEF), the Office of the High Commissioner for Human Rights (OHCHR), the United Nations Programme for Gender Equality and the Empowerment of Women (UN Women), the United Nations Educational, Scientific and Cultural Organization (UNESCO) and WHO reaffirmed their commitment to eliminating female genital mutilation and ensuring survivors receive quality, appropriate services. 

Female genital mutilation is a violation of human rights and cannot be justified on any grounds. It compromises girls’ and women’s physical and mental health and can lead to serious, lifelong complications.

 The statement highlights accelerating progress: half of all global gains since 1990 occurred in the past decade, reducing prevalence from one in two girls to one in three. Yet leaders warned that funding cuts, declining investment in health and education, and growing pushback, including dangerous claims that female genital mutilation is acceptable when performed by medical personnel, threaten hard‑won progress. They emphasized proven strategies such as community‑led movements, education, engaging religious and community leaders, and comprehensive support for survivors. 

https://bit.ly/46alXEz

## Oral cholera vaccine 

Global supply of oral cholera vaccine is now sufficient to restart preventive vaccination campaigns after more than three years of shortages, Gavi the Vaccine Alliance, UNICEF and WHO announced. An initial 20 million doses are being deployed: 3.6 million to Mozambique, 6.1 million to the Democratic Republic of the Congo and 10.3 million planned for Bangladesh.

Following sustained efforts by global agencies, manufacturers and partners, annual global supply has doubled from 35 million doses in 2022 to nearly 70 million in 2025, enabling the restoration of preventive campaigns. Agencies emphasized that vaccination must be paired with long‑term investment in water, sanitation and resilient health systems to reduce cholera risk. 

“The multi-year surge in cholera cases and resulting unprecedented demand for vaccines were stark reminders that sustainable, accessible vaccine supply is a global public good, and the world cannot afford complacency,” said Sania Nishtar, chief executive officer of Gavi, the Vaccine Alliance. 

The three countries were chosen based on allocation criteria set out by the Global Task Force for Cholera Control (GTFCC), a partnership of over 50 organizations, to ensure cholera vaccines for preventive campaigns are distributed systematically, equitably and transparently.

“This milestone shows the power of bringing together diverse partners to build a more reliable response to cholera. Preventive vaccination helps shield communities and buys critical time. However, lasting progress will depend on long‑term investment in infrastructure, for which political commitment is indispensable,” said Ilesh Jani, chair of the Steering Committee of the GTFCC.

https://bit.ly/4tNE7X0

## Preventable cancer assessment

Up to four in ten cancer cases worldwide could be prevented, according to a new global analysis from WHO and the International Agency for Research on Cancer (IARC). Released ahead of World Cancer Day, the study estimates that 37% of all new cancer cases in 2022, around 7.1 million, were linked to preventable causes. Drawing on data from 185 countries and 36 cancer types, it identifies tobacco as the leading preventable cause of cancer. Alcohol use and infections with hepatitis viruses and human papillomavirus are also leading causes. Lung, stomach and cervical cancers accounted for nearly half of all preventable cases globally.

The analysis highlights major differences between men and women, with preventable cancers making up 45% of cases in men and 30% in women. Regional disparities were also significant, reflecting variations in exposure to behavioural, environmental and infectious risks. “This is the first global analysis to show how much cancer risk comes from causes we can prevent,” said André Ilbawi, WHO team lead for cancer control. “By examining patterns across countries and population groups, we can provide governments and individuals with more specific information to help prevent many cancer cases before they start.”

The findings underscore the need for strong tobacco control, alcohol regulation, vaccination against cancer‑causing infections, cleaner air, safer workplaces and healthier environments to reduce the global cancer burden. 

https://bit.ly/4rqztwp

## Onchocerciasis in Yemen

Yemen has achieved a breakthrough in the fight against onchocerciasis, or “river blindness,” reaching remote high‑altitude communities long deprived of treatment. In 2025, under the leadership of the Ministry of Public Health and Population and with WHO’s technical support, the country launched one of its most ambitious mass drug administration campaigns, targeting districts historically considered unreachable due to conflict, rugged terrain and chronic funding gaps.

 A redesigned door‑to‑door strategy enabled health teams to reach remote areas, achieving full geographical access and surpassing WHO’s recommended 80% coverage threshold, with 91% coverage in Hajjah and Al‑Mahweet governorates and 86.5% in Taiz.

 Community leadership was central to the campaign’s success. More than 400 local volunteers, over half of them women, administered more than 732 000 donated doses of ivermectin, building trust and reaching households for the first time. 

These efforts demonstrate that elimination is possible even in conflict‑affected settings when adaptive strategies, community engagement and sustained partnerships come together. Continued investment remains essential to keep Yemen on track to eliminate onchocerciasis by 2030. 

https://bit.ly/3MvNDgy

**Cover photo**

People collecting water from a riverbed in Purulia, West Bengal, India. Due to climate change, the monsoon season is becoming more irregular, which is causing rivers to dry out.

**Figure:**
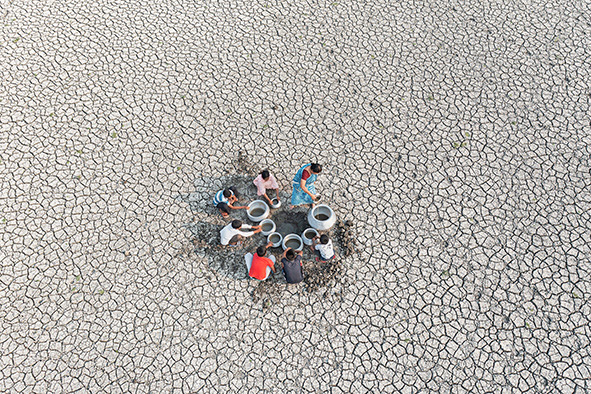
Sandipani Chattopadhyay / Wellcome Photography Prize 2025

**Looking ahead**

9–12 March. Strategic Advisory Group of Experts on Immunization (SAGE), online meeting. https://bit.ly/3OMP0bc

24 March. World TB Day 2026, global events. https://bit.ly/4riyle4

